# Dynamic RNA profiling in *Plasmodium falciparum *synchronized blood stages exposed to lethal doses of artesunate

**DOI:** 10.1186/1471-2164-9-388

**Published:** 2008-08-18

**Authors:** Onguma Natalang, Emmanuel Bischoff, Guillaume Deplaine, Caroline Proux, Marie-Agnès Dillies, Odile Sismeiro, Ghislaine Guigon, Serge Bonnefoy, Jintana Patarapotikul, Odile Mercereau-Puijalon, Jean-Yves Coppée, Peter H David

**Affiliations:** 1 Institut Pasteur, Unité d'Immunologie Moléculaire des Parasites; CNRS URA 2581, 28 Rue du Docteur Roux, F-75724, Paris, Cedex 15, France; 2 Institut Pasteur, Plate-Forme 2 – Puces à ADN, 28 Rue du Docteur Roux, F-75724, Paris, Cedex 15, France; 3 Faculty of Tropical Medicine, Mahidol University, 420/6 Rajvithi Road, Bangkok, 10400, Thailand

## Abstract

**Background:**

Translation of the genome sequence of *Plasmodium sp*. into biologically relevant information relies on high through-put genomics technology which includes transcriptome analysis. However, few studies to date have used this powerful approach to explore transcriptome alterations of *P. falciparum *parasites exposed to antimalarial drugs.

**Results:**

The rapid action of artesunate allowed us to study dynamic changes of the parasite transcriptome in synchronous parasite cultures exposed to the drug for 90 minutes and 3 hours. Developmentally regulated genes were filtered out, leaving 398 genes which presented altered transcript levels reflecting drug-exposure. Few genes related to metabolic pathways, most encoded chaperones, transporters, kinases, Zn-finger proteins, transcription activating proteins, proteins involved in proteasome degradation, in oxidative stress and in cell cycle regulation. A positive bias was observed for over-expressed genes presenting a subtelomeric location, allelic polymorphism and encoding proteins with potential export sequences, which often belonged to subtelomeric multi-gene families. This pointed to the mobilization of processes shaping the interface between the parasite and its environment. In parallel, pathways were engaged which could lead to parasite death, such as interference with purine/pyrimidine metabolism, the mitochondrial electron transport chain, proteasome-dependent protein degradation or the integrity of the food vacuole.

**Conclusion:**

The high proportion of over-expressed genes encoding proteins exported from the parasite highlight the importance of extra-parasitic compartments as fields for exploration in drug research which, to date, has mostly focused on the parasite itself rather than on its intra and extra erythrocytic environment. Further work is needed to clarify which transcriptome alterations observed reflect a specific response to overcome artesunate toxicity or more general perturbations on the path to cellular death.

## Background

Efforts at extracting biologically meaningful information from the genome sequence of *Plasmodium sp*. are fueled by the necessity to find new methods of malaria control. *Plasmodium falciparum *resistance to most common antimalarials such as chloroquine, sulfadoxine or pyrimethamine, is now-days widespread, and resistance to other antimalarials is increasing alarmingly. There is recent evidence for emerging in vitro resistance to one of the major groups of drugs, artemisinins [1] and treatment failures to recently introduced drug combinations including artemisinin derivatives [2,3]. Research priorities include the discovery of new drugs, the understanding of drugs' mode of action and resistance mechanisms. Whatever the drug's mode of action, a better appraisal of the biological processes taking place in a parasite dying within an erythrocyte is needed to uncover additional drug targets and potentiate the effect of existing weapons. Towards reaching such goals, much hope rests on high through-put technologies such as DNA microarrays aimed at the study of gene expression at the genome scale and proteomics. Both approaches are complementary, and each has its own limitations. Proteomic approaches are complicated by solubility issues, detection of low abundance proteins and post-translational alterations. Both proteome and transcriptome approaches investigate steady state levels, a net sum of synthesis and decay of the gene product. Transcriptome studies are limited by the uncertain correlation of steady state RNA with protein levels. They nevertheless deliver a comprehensive and sensitive, genome-wide exploration of expression profiling. This proved invaluable in deciphering the developmental cycle gene expression patterns in malaria parasites [4,5].

Few studies have shown that the transcriptome of *P. falciparum *can be altered by exposure of the parasite to a drug. Analysis of the parasite transcriptome under doxycyclin showed that apicoplast gene expression was deeply altered [6]. This study is so far the only published example of a specific transcriptome response to a drug related to the drug's known target-pathway. No significant changes of the mRNA levels for enzymes involved in the lipid biosynthesis pathway could be evidenced upon exposure for 24–36 hours to the antimalarial choline analog T4, which targets the inhibition of phosphatidyl choline biosynthesis [7]. In a recent study of the transcriptome of asynchronous *P. falciparum *cultures under chloroquine pressure [8], around 600 genes were differentially expressed in presence of the drug, but only 38 of these were observed in two different experiments. Such poor reproducibility of results along with the low expression ratios observed for these 38 genes was interpreted as reflecting limited reactivity of the parasite transcriptome to environmental stimuli and the possible importance of post-transcriptional gene expression regulation in *Plasmodium*. However, the observed discrepancies may relate to different proportions of developmental stages in the 2 populations explored. Indeed, parasite maturation stage is an essential variable to control, as a majority of *P. falciparum *genes are differentially expressed during the 48 hour erythrocytic cycle [4]. Such developmentally regulated control of gene expression hinders the interpretation of results if drug treatment exerts a retardation effect on the parasite erythrocytic cycle.

We have used here a novel strategy and analysed the alterations of the parasite transcriptome in synchronous parasite cultures shortly after exposure to a lethal dose of artesunate, a rapidly acting drug. Artemisinin derivatives are potent antimalarials that are the cornerstone of currently recommended drug combination to treat *P. falciparum *malaria [9]. Artemisinins, which are endoperoxide-containing sesquiterpene lactones are structurally distinct from all other anti-malarials. Importantly they are the most rapidly acting antimalarials known to date, which moreover are active on young as well mature erythrocytic stages [10]. These unique properties allowed the kinetic analysis of the events occurring in synchronous cultures of parasites exposed to a lethal drug concentration at different time points along the intraerythrocytic developmental cycle. We explored the dynamics of transcriptome alteration during the process leading to parasite death in different parasite stages. This identified 398 genes with dynamic transcriptome alterations after exposure to artesunate.

## Results

### Choice of experimental design

In order to work with homogeneous parasite populations, tightly synchronized parasites were used in all experiments, along with an artesunate concentration which irreversibly damages 100% of the parasites within 3 hours. The lethal dose of artesunate, defined here as the minimal concentration needed for a 3 hour exposure to completely prevent reinvasion (see "Materials and methods" section), was 780 nM. Importantly, it corresponds to 100–1000 times the IC_50_, this dose remains physiological, being in the range of peak plasma concentration reached in patients administered artesunate (1300–8400 nM) [11].

Each experiment consisted in an artesunate-free culture (control) and an artesunate treated culture. For each time point explored, cDNA from parasites with and without the drug were labeled with different cyanines, mixed and hybridized. To analyse dynamic transcriptome alterations, 2 pilot experiments were performed in which RNA was harvested at 30, 90, 180 minutes and 10 hours of incubation with the drug. No significant changes in gene expression could be detected at 30 minutes. In contrast, after 10 hour incubation time, major transcriptome alterations occurred under artesunate, but their analysis was un-interpretable due to drug-induced massive slowing-down of parasite development (data not shown). We thus decided to focus on 2 drug-exposure time, namely 90 minutes and 3 hours after drug addition.

Since artesunate is active on ring stages as well as on older trophozoite stages, we decided to explore different time points along the erythrocytic cycle and search for genes affected at each time point. Therefore, 5 drug treatment experiments, staggered between 20 hours and 30 hours of parasite development, were performed for the comparison of drug versus no drug at 90 minutes and 3 hours.

### Transcript changes under artesunate exposure

We looked for genes displaying similar changes across all experiments, with the hypothesis that responses to artesunate would not be stage-dependent within the explored 10 hour window of the developmental cycle. The results of 5 experiments performed at time points staggered between 20–30 hours post-invasion, were analyzed using ANOVA with a Holm-Bonferroni method of p-value adjustment, p < 0.05. This led to a list of genes with similar ratios of expression (i. e. low variance). From this list, only genes differentially expressed at 3 hours under artesunate and not after 3 hours in the absence of the drug were retained (p = 1). It is important to stress that what we refer to, for convenience, as over- or under-expression in fact refers to higher or lower steady state RNA levels.

Due to the high number of microarrays analyzed (32 at 3 hours), power of the statistical analysis was such that expression log ratios as low as +/- 0.3 could be found for genes that displayed significant changes by ANOVA [for distribution of log ratios, see Additional file 1]. This appeared questionable from a biological standpoint and we chose to use a cut-off log-ratio of +/- 0.8. This value is lower than what is usually (and often arbitrarily) chosen in most transcriptome studies, i.e. +/- 1, but we felt it was justified given the large amount of data analyzed here and the low amplitude of fold-changes reported in previous *P. falciparum *transcriptome studies [8,12].

In addition, as genes of potential interest could be differentially expressed in the controls but more-so or differently under artesunate, we needed to identify these and filter out the developmentally regulated gene expression profiles. Indeed, the extent to which the drug slowed-down parasite development is unknown. Thus, different expression levels in artesunate-exposed vs. control (unexposed) cultures could reflect the direct effect of artesunate together with a possible difference in growth rate/developmental stage. To identify the developmentally regulated genes in the artesunate-free control culture, the 3 hours RNA was hybridized against the time 0 RNA (3 control experiments). Even within such a short time window, 81 genes showed differential expression in the control culture. Filtering-out this potential confounding "slowing-down effect" established a second list of genes differentially expressed in the artesunate experiments and in the control experiments, but with significantly different expression-ratios under these two conditions (p < 0.05) [see Additional file 2 for the list of differentially expressed genes].

This resulted in the following: out of the 4703 genes analyzed, 398 (approx 8.5%) were differentially expressed after 3 hours in presence of artesunate, 244 over-expressed (approx 5%) and 154 (approx 3.5%) under-expressed. Of these 398 genes differentially expressed at 3 hours, 42 showed significant expression changes at 90 minutes, presumably reflecting an early response to the drug.

Our technically demanding and relatively stringent approach may have generated false negatives, but we wanted to be as confident as possible about the list of genes selected as being differentially expressed. This confidence was supported by the results of qRT-PCR performed with 12 over-expressed and 4 under-expressed genes, which confirmed the microarray data (Spearman correlation coefficient: 0.81) [see Additional file 3 for the comparison between microarray and qRT-PCR results].

### Classification of differentially expressed genes

**Figure 1:**
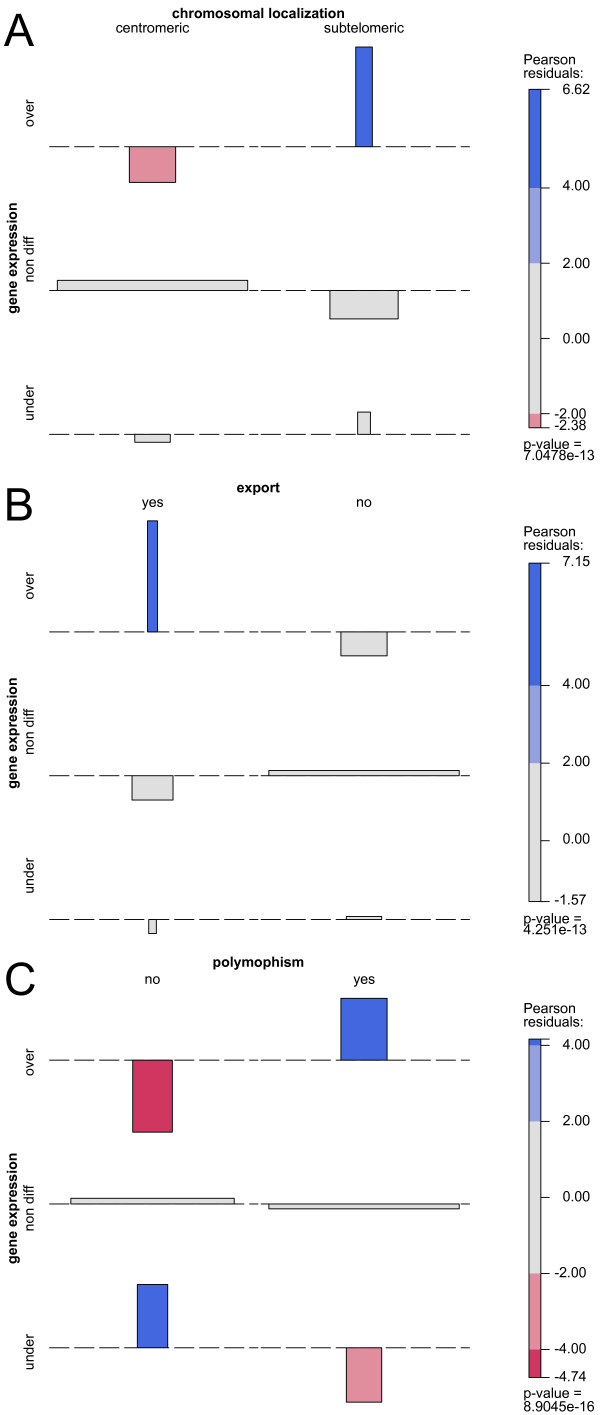
**Association plots of gene expression with gene chromosomal localization, predicted export sequences and polymorphism**. A : Bias in gene expression versus gene chromosomal localization (subtel = subtelomeric position of gene defined as <150 kb from telomere). B: Bias in gene expression versus presence of predicted export signals [13]. C: Bias in gene expression versus gene polymorphism (as defined by presence or absence of non synonymous SNPs, from analysis of 3D7, Dd2, HB3, Ghana1 and IT genomes). Bar width is proportional to the number of genes per category and bar height to the Pearson residuals for an independence model. Blue and red colors indicate significant bias.

Interesting features were observed in the group of differentially expressed genes. For genes expressed differentially at 3 hours, there was a strong positive bias in the sub-set of over-expressed genes towards polymorphic genes located in sub-telomeric position and carrying PEXEL/HCT motifs [13] used for export beyond the parasitophorous vacuole into the erythrocyte cytoplasm, or to the red blood cell membrane/surface (Figure 1). This was determined after excluding from the analysis the strain-specific *var, stevor *and *rifin *multigene families – which are not adequately represented in our case since the parasite strain used differs from 3D7. Concomitant with this positive bias, there was a negative bias towards conserved genes in the over-expressed genes. In contrast, the group of under-expressed genes displayed a positive bias towards conserved genes and a negative bias towards polymorphic genes.

**Figure 2:**
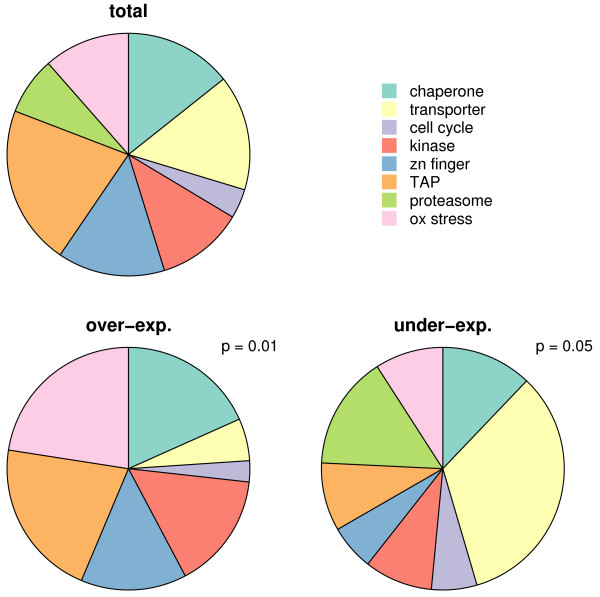
**Differential gene expression under artesunate exposure: transcript distribution among different gene functional categories**. "Total" pie-chart: genes present in the 8 different color-coded functional categories as distributed in the genome represented on the DNA array. "over-exp." and "under-exp." pie-charts: distribution of over-expressed and under-expressed genes respectively, among the 8 different functional categories. p-value: p-value of chi-square test of over-expressed gene and under-expressed gene compared to the total genes in the genome represented on the DNA array.

Functions (in most cases putative) were assigned to 213 of the 398 differentially expressed genes [12,14-21] [see Additional file 2]. Apart from lipid and purine/pyrimidine metabolism, few genes belonged to the metabolic pathways of the parasite (as defined by Ginsburg [16]). Most genes with altered expression under artesunate pressure were related to chaperones, transporters, cell cycle, kinases, Zn finger proteins, transcription activating proteins, proteins involved in proteasome degradation, oxidative stress and in cell cycle regulation. A positive bias towards over-expression could be observed for genes related to oxidative stress, kinases, transcription associated proteins and chaperones, and towards under-expression for genes related to proteasome degradation and transporters (Figure 2). Slowing down of parasite development may be linked to the differential expression of three genes involved in cell cycle regulation [see Additional file 4]. Two genes were over-expressed, namely PFF1125 encoding a putative RNA binding protein mei2 homologue and PFL1855 encoding a putative cell cycle control protein, while PFA0345w, encoding a putative centrin, was under-expressed.

### Oxidative stress

Oxidative stress is likely to occur in parasites submitted to artesunate, whether linked to the direct action of the drug or to the reduced capacity of the damaged parasite to inactivate free radicals. The antioxidant defense in *P. falciparum *involves both the glutathione and the thioredoxin system, the relative contribution of each remaining unknown [22]. Four genes involved in the parasite antioxidant defense [22,23], showed altered transcription profiles (Table 1) : gamma glutamyl cystein synthetase and glutathione synthetase (both involved in the glutathione system) were over-expressed. In contrast, the genes encoding protein disulfide isomerase related protein (potentially involved in oxidative protein folding, catalyzing both the oxidation and isomerization of disulfides on nascent polypeptides [24]) and glutathione peroxidase (shown to rather encode a thioredoxin peroxidase [25] and thus involved in the thioredoxin system) were under-expressed. The consecutive action of gamma glutamyl synthetase and glutathione synthetase leads to the production of reduced glutathione (GSH), which plays a pivotal role in the antioxidant defense through maintenance of the red-ox state of protein -SH moeties, the reduction of the noxious hydrogen and lipid peroxides and the extrusion of toxic compounds (including drugs) [22]. The predicted outcome of the over-expression of these 2 genes would be an increased supply of GSH in response to the oxidative stress induced by artesunate.

**Table 1:** Differentially expressed genes involved in antioxidant defence in artesunate-treated cells

**Gene ID**	**Description**	**ART 90 min**	**ART 3 h**	**HS**	**CQ**
PFI0925w	gamma-glutamylcysteine synthetase		1,41	+	
PFE0605c	glutathione synthetase		0,94		
PFL0595c	glutathione peroxidase		-0,95		
PF11_0352	protein disulfide isomerase related protein		-0,89	-	

Genes coding for proteins involved in antioxidant defence defined in [23].

Gene ID: identifier as found in PlasmoDB.

Art columns: log ratios of gene expression under artesunate exposure at 90 minutes and 3 hours.

HS column: over (+) or under (-) expression.

Results were compared with transcriptome modifications induced by heat-shock (HS) [12] and chloroquine (CQ) [8].

### Chaperones, protein trafficking and related genes

Genome mining on parasite chaperone analogues has led to the conclusion that approximately 2% of the *P. falciparum *genes encoded potential or confirmed chaperones [19]. Chaperone proteins have diverse functions, such as serving as transcription factors, regulating the cell cycle, being involved in protein degradation, binding damaged proteins, protein transport across membranes and protein folding. As expected in a parasite under severe injury, 13 genes encoding chaperone or chaperone-related proteins (Table 2) were found up-regulated at 3 hours of artesunate pressure, among which PfHsp70, PfHsp90 and 6 genes belonging to the Hsp40 chaperone machinery of *P. falciparum *[20]. The first four were already over-expressed at 90 minutes, along with PFD0080c (a member of the *resa*-like DnaJ family) and thus can be considered part of the early response to the drug. Interestingly, most (9/13), chaperone-encoding genes up-regulated under artesunate have potential export signals, suggesting possible export of their corresponding proteins from the parasite to the erythrocyte cytoplasm or membrane. Chaperone involvement in trafficking of membrane or exported proteins has been highlighted in a recent analysis of the parasite chaperone network [21]. In particular, analysis of the interactome suggested that a member of the Hsp40 family (PF14_0700), which interacts with PfHsp70 (PF08_0054) and PfHsp90 (PF07_0029) could function as a co-chaperone in the Hsp90 complex of the parasite to transport eythrocyte membrane exported proteins such as Antigen 332 (PF11_0507). It is remarkable to note that PF14_0700, PF08_0054, PF07_0029 and PF11_0507 were all over-expressed under artesunate pressure [see Additional file 2]. This suggests artesunate-triggered altered intracellular trafficking, contributing to remodeling of the parasitized erythrocyte membrane, a conclusion further substantiated by the observed increased steady state levels of genes encoding proteins interacting with the erythrocyte cytoskeleton such as MESA (PFE0040c), several members of the RESA family and number of genes carrying PEXEL/HT motifs. Alterations of the intracellular trafficking is also suggested by altered transcription profiles of genes such as signal peptidase (MAL13P1.167) as well as transporters, including organellar importers (see below).

**Table 2:** Differentially expressed chaperone and chaperone-related genes in artesunate-treated cells

**Gene ID**	**Description**	**Pexel**	**family**	**ref**	**ART 90 mn**	**ART 3 h**	**HS**	**CQ**
PFE1605w	DNAJ protein	yes	resa like/dnaJ	1, 3		1,01		
PFD0080c	hypothetical protein, conserved in P.falciparum	yes	resa like	3	1,31	1,82	+	
PF10_0378	DnaJ protein, putative	yes	Hsp40, type III	1, 2, 4	1,33	1,53	+	
PFE0040c	mature parasite-infected erythrocyte surface antigen (MESA) or PfEMP2	yes	Hsp40, type IV	2, 4	0,92	1,87		
PFA0660w	protein with DNAJ domain, dnj1/sis1 family	yes	Hsp40, type II	1, 2, 4	1,01	1,34	+	
MAL7P1.7	RESA-like protein	yes	resa like	3		0,83		
PFL0050c	hypothetical protein	yes	resa like	3		0,86		
PF07_0029	heat shock protein 86	no	hsp90	1		0,9	+	
PF07_0030	heat shock protein 86 family protein	no	Hsp86 family	5		1,63	+	
PF08_0054	heat shock 70 kDa protein	no	hsp70	1		0,88	+	
PF11_0034	hypothetical protein	yes	Hsp40, type IV	1, 2, 4		0,81	+	
PFA0675w	P. falciparum RESA-like protein with DnaJ domain	yes	Hsp40, type IV	1, 2, 4		1,96	+	
PF14_0700	hypothetical protein, conserved	no	Hsp40, type III	1, 2, 4		0,81		
PF11_0509	ring-infected erythrocyte surface antigen putative	yes	Hsp40, type IV	1, 2, 4		-1,14		
PF13_0102	DNAJ-like Sec63 homologue	no	Hsp40, type III	1, 2, 4		-1,04		
PF11_0216	hypothetical protein	no	Heat shock factor binding protein 1	6		-0,93	-	
PFC0975c	PFCYP19, cyclophilin, peptidyl-prolyl cis-trans isomerase	no	cyclophillin	1		-0,97		

Gene ID: identifier as found in PlasmoDB.

Art columns: log ratios of gene expression under artesunate exposure at 90 minutes and 3 hours.

HS column: over (+) or under (-) expression.

Results were compared with transcriptome modifications induced by heat-shock (HS) [12] and chloroquine (CQ) [8].

Chaperone- and chaperone-related genes were defined based on: 1) Acharya et al. [19], 2) Botha et al. [20], 3) resa-like annotation from GeneDB, 4) genes fitting the HMM profile of DNAJ (PF00226) from Pfam, 5) The Hsp86 family annotation from GeneDB, 6) genes fitting the HMM profile of heat shock factor binding protein 1 (PF06825) from Pfam.

### Transporters

Eleven genes involved in transport were under-expressed (Table 3). The putative UDP galactose antiporter (PF11_0141) down-regulated at 3 hours, was the only transporter found down-regulated at 90 minutes, suggesting that alteration of its transcript levels is an early event. Four subunits of the vacuolar ATP synthase were under-expressed. This constitutes one of the few cases of concommittant regulation of individual components of a specific machinery or pathway in this study, suggesting that indeed the activity of the vacuolar ATP synthase was severely impaired. This enzyme contributes to acidification of vacuolar and organelle contents.

**Table 3:** Differentially expressed transport associated genes in artesunate-treated cells

**Gene ID**	**Description**	**ART 90 min**	**ART 3 h**	**HS**	**CQ**
PF07_0121	NMD3 protein, putative		0,85		
PFB0435c	amino acid transporter, putative		0,89		
PFE1455w	sugar transporter, putative		0,92		+
PFE1150w	multidrug resistance protein, *Pfmdr1*		1,10		
PF11_0141	UDP-galactose transporter, putative	-0,86	-0,82	-	
PFC0725c	formate-nitrate transporter, putative		-1,16	-	
MAL8P1.13	integral membrane protein, conserved/folate-Biopterin transporter		-1,13		
MAL13P1.206	Na+ -dependent Pi transporter, sodium-dependent phosphate transporter *PfPiT*		-0,90		
PF13_0252	nucleoside transporter 1 *PfNT1/PfENT1*		-1,32		
PFL0170w	Transporter, major facilitator superfamily		-0,86	-	
PFE0410w	triose or hexose phosphate/phosphate translocator, putative		-0,88	-	
PF13_0227	vacuolar ATP synthase subunit D, putative		-0,92		
PF11_0412	Vacuolar ATP synthase subunit F, putative		-0,81	-	
PF13_0130	vacuolar ATP synthase subunit G, putative		-0,82	-	
PFE0965c	vacuolar ATP synthetase, subunit C putative		-0,85		

Gene ID: identifier as found in PlasmoDB.

Art columns: log ratios of gene expression under artesunate exposure at 90 minutes and 3 hours.

HS and CQ columns: over (+) or under (-) expression.

Results were compared with transcriptome modifications induced by heat-shock (HS) [12] and chloroquine (CQ) [8].

Genes involved in transport were selected from the pathway maps of Ginsburg [16].

Only 4 genes encoding transporters were over-expressed, amongst which *pfmdr1 *(PFE1150w), the P-glycoprotein homologue of *P. falciparum*, which modulates susceptibility to antimalarial drugs such as quinine, mefloquine and artemisinins [26-28]. An association has been shown in the field between sensitivity to arylaminoalcohols or endoperoxides, *pfmdr1 *allelic status and gene copy number [29], suggesting that an over-expressed functional *pfmdr1 *conferred a multidrug resistance like phenotype [30,31]. Over-expression of *pfmdr1 *we show to occur under artesunate pressure may have an impact on susceptibility to the drug(s) associated to artemisinins upon ACT administration. It also suggests that not only gene copy number but also gene transcription rates and mRNA stability should be monitored when exploring mechanisms of field parasite drug resistance.

### Lipid metabolism and the Apicoplast

Eight genes involved in lipid metabolism were differentially expressed (Table 4).

**Table 4:** Differentially expressed genes involved in lipid metabolism in artesunate-treated cells

**Gene ID**	**Description**	**ART 90 min**	**ART 3 h**	**HS**	**CQ**
MAL13P1.485	acyl-coa ligase antigen		0,94		
PFB0685c	acyl-CoA synthetase, PfACS9		0,83		
PFB0695c	acyl-CoA synthetase	0,88	1,02	+	
PF14_0761	fatty acyl CoA synthetase 1 PfACS1		0,99		
PF14_0664	biotin carboxylase subunit of acetyl CoA carboxylase, putative	0,97	1,31	+	
PF08_0099	acyl CoA binding protein, putative		-0,89		
PF10_0016	acyl CoA binding protein, putative		-1,04	-	
PFE0410w	triose or hexose phosphate/phosphate translocator, putative		-0,88	-	

Gene ID: identifier as found in PlasmoDB.

Art columns: log ratios of gene expression under artesunate exposure at 90 minutes and 3 hours.

HS column: over (+) or under (-) expression.

Results were compared with transcriptome modifications induced by heat-shock (HS) [12] and chloroquine (CQ) [8].

Genes were selected based on [16].

*P. falciparum *contains an unusually high number of Acyl CoA synthases (ACS) and binding proteins that might play a role in fatty acid salvage and transport from the host cell [32,33]. Amongst the 13 different ACS genes, a non subtelomeric acyl CoA synthetase gene PFB0685c has expanded into a family of 9 duplicated genes mainly located in the subtelomeric regions of the genome and transcribed during the erythrocytic stage of the parasite. At least two ACS proteins PfACS1 (PF14_0761) and PfACS3 (PFL2570w) were shown to interact with ankyrin through their lysine-rich C-terminal domain [34]. Similar domains are only present in the genes of this family, possibly targeting these enzymes to the erythrocyte cytoplasm and/or membrane, an interesting possibility as all the ACS proteins have a signal peptide but are devoid of the PEXEL/HT motif. This would contribute to the capacity of *P. falciparum *to activate fatty acid scavenged from the plasma or the erythrocyte membrane, a critical metabolic function for the survival of the parasite. All 4 ACS genes over-expressed under artesunate (PFB0685c, PFB695c, MAL13P1_485 and PF14_0761) belong to this family.

Two acyl-CoA binding protein genes, PF08_0099 and PF10_0016, were under-expressed. PF14_0664, an Acetyl CoA carboxylase which generates Malonyl-CoA, likely to be located in the apicoplast [32], was already found over-expressed at 90 minutes. Interestingly, the triose phosphate transporter located in the outermost membrane of the apicoplast, PFE0410w, was under-expressed under artesunate. This transporter, also called PFoTPT, fuels the apicoplast by providing carbon, reducing power and ATP [35], but also quite importantly, dihydroxyacetone phosphate (DHAP), the precursor needed for isoprenoid and for phospholipids biosynthesis [36]. Altogether these data suggest an alteration of fatty acid metabolism both intracellularly and in the apicoplast.

### Mitochondrial genes and genes encoding proteins targeted to the mitochondrion

The main metabolic function allowed by the active mitochondrial electron transport chain maintained by *Plasmodium *is regeneration of ubiquinone, which is required as the electron acceptor for dihydroorotate dehydrogenase, an essential enzyme for pyrimidine biosynthesis [37]. Indeed, 3 important genes involved in mitochondrial electron transport are under-expressed in presence of artesunate: 2 subunits of the cytochrome c oxydase (downstream of the cytochrome bc1 complex inhibited by atovaquone) coxI and coI and ubiquinol-cytochrome c reductase hinge protein, PF14_0248 (Table 5). This interference with the electron transport chain could in turn affect pyrimidine biosynthesis.

**Table 5:** Differentially expressed genes related to the mitochondrion in artesunate treated cells

**Gene ID**	**Description**	**ART 90 min**	**ART 3 h**	**HS**	**CQ**
PF08_0054	heat shock 70 kDa protein		0,88	+	
coxI	putative cytochrome oxidase I		-1,07		
coI	putative cytochrome oxidase I		-1,15		
PF14_0248	ubiquinol-cytochrome c reductase hinge protein, putative		-0,99	-	
PFC0975c	PFCYP19, cyclophilin, peptidyl-prolyl cis-trans isomerase		-0,97		

Gene ID: identifier as found in PlasmoDB.

Art columns: log ratios of gene expression under artesunate exposure at 90 minutes and 3 hours.

HS column: over (+) or under (-) expression.

Results were compared with transcriptome modifications induced by heat-shock (HS) [12] and chloroquine (CQ) [8].

Genes were selected based on [16].

PFC0975c encoding a peptidyl-prolyl cis-trans isomerase cyclophilin-type belonging to the chaperone network of the mitochondrial matrix [16] is under-expressed in response to artesunate. Cyclophilin D/peptidylprolyl isomerase activity is a regulator of mitochondrial permeability transition, a non selective inner-membrane permeabilization occuring in response to increased calcium load and redox stress. These 2 conditions are induced by artesunate. Such a phenomenon can lead to necrosis through activation of phospholipases, proteases and nucleases [38].

### Purine/pyrimidine metabolism

In addition to the possibly impaired respiratory chain interfering with pyrimidine synthesis, expression of other genes related to purine/pyrimidine biosynthesis was also modified by artesunate pressure (Table 6): cytidine and deoxycytidylate deaminase (PF13_0259) hypoxanthine phophorybosyl transferase (PF10_0121), deoxyuridine 5'triphosphate nucleotidohydrolase (PF11_0282) and uridine phosphorylase (PFE0660c) are all under-expressed, while dihydroorotase (PF14_0697) is over-expressed. Interference with such key metabolic processes may readily cause parasite death.

**Table 6:** Differentially expressed genes related to purine/pyrimidine metabolism in artesunate-treated cells

**Gene ID**	**Description**	**ART 90 min**	**ART 3 h**	**HS**	**CQ**
PF14_0697	dihydroorotase, putative	0,96	0,93		
PF13_0259	cytidine and deoxycytidylate deaminase family, putative		-1,22		
PF10_0121	hypoxanthine phosphoribosyltransferase		-1,24		
PF11_0282	deoxyuridine 5'-triphosphate nucleotidohydrolase, putative	-0,88	-0,95		
PFE0660c	purine nucleotide phosphorylase, putative	-1,07	-1,53		

Gene ID: identifier as found in PlasmoDB.

Art columns: log ratios of gene expression under artesunate exposure at 90 minutes and 3 hours.

Results were compared with transcriptome modifications induced by heat-shock (HS) [12] and chloroquine (CQ) [8].

Genes were selected based on [16].

### Signaling/kinases

The kinome of *P. falciparum *is atypical, with an atypical MAP kinase family and a particular R45-FIKK kinase family [39].

Fourteen genes encoding kinases, some of which potentially related to signal transduction, were differentially expressed in presence of artesunate (Table 7). Four of the 11 over-expressed genes were already differentially expressed at 90 minutes, among which the calcium calmodulin dependent protein kinase 2, PfPK2 (PFL1885c). PfPK2 belongs to the CamK group of *P. falciparum *kinases, a family of kinases which can be activated in response to increased calcium levels, possibly resulting from specific inhibition of the SERCA PfATPase6 by the drug [40]. The other 3 genes with increased expression levels at 90 minutes were members of the R45-FIKK multigene family [18,41]. This Apicomplexa-specific kinase gene family is composed of 20 genes all presenting sub-telomeric positions, scattered over 11 chromosomes. It has recently been shown that these kinases localize in different compartments of the infected erythrocyte, some being associated with the erythrocyte membrane [42]. Under artesunate pressure, 3 members of the R45-FIKK family were over-expressed at 90 minutes (PFI0095c, MAL7P1.144 and PFL0040c) and remained at elevated levels subsequently, while two additional R45-FIKK Kinases displayed increased transcript levels at 3 hours, namely PFD1165w and PF11_0510. All 5 present export motifs. PFD1165w and PFL0040c are associated with Maurer's clefts, and PFL0040c with the infected erythrocyte membrane as well [42].

**Table 7:** Differentially expressed genes encoding kinases in artesunate-treated cells

**Gene ID**	**Description**	**Kinase type**	**Name**	**ART 90 min**	**ART 3 h**	**HS**	**CQ**
PFL0040c	protein kinase, R45/FIKK family	R45/FIKK	FIKK12	0,88	1,16		
MAL7P1.144	protein kinase, R45/FIKK family	R45/FIKK	FIKK7.1	0,90	1,82		
PFI0095c	protein kinase, R45/FIKK family	R45/FIKK	FIKK9.1	0,83	1,28		
PF11_0510	protein kinase, R45/FIKK family	R45/FIKK	FIKK11		0,95		
PFD1165w	protein kinase, R45/FIKK family	R45/FIKK	FIKK4.1		1,05		
PFL1885c	calcium/calmodulin-dependent protein kinase 2, putative	kinase	PfPK2	1,35	1,21		
PFL2280w	cyclin g-associated kinase, putative	kinase			1,10		
PF14_0346	cGMP-dependent protein kinase 1, beta isozyme, putative	kinase			1,03		
PF11_0242	protein kinase	kinase			0,95		
PF14_0294	mitogen-activated protein kinase 1, PfMAP1	kinase	PfMAP1		1,05		
PFD0975w	ROI kinase-like protein	RIO1			0,90		+
PF11_0377	casein kinase 1, PfCK1	kinase			-0,91		+
PF11_0227	serine/threonine protein kinase, puative	kinase			-0,98		
PFB0150c	protein kinase, putative	kinase			-1,05		

Gene ID: identifier as found in PlasmoDB.

Art columns: log ratios of gene expression under artesunate exposure at 90 minutes and 3 hours.

CQ column: over (+) or under (-) expression.

Results were compared with transcriptome modifications induced by heat-shock (HS) [12] and chloroquine (CQ) [8].

Kinases were defined as genes fitting the HMM profil kinase PF00069 (update of Ward et al. [39]) and ROI Kinases as genes fiiting the HMM profil PF01163. FIKK kinases were defined as described in Schneider and Puijalon [41].

### Transcription associated proteins; zinc finger genes

Transcriptome studies of parasites during the erythrocytic stages of development have shown that in many cases, transcription is soon followed by translation, when the parasite "needs" the considered proteins to further develop [4]. In contrast, comparison between transcriptomic and proteomic studies have shown the existence of discrepancies in RNA and protein expression for about 50% of the genes expressed during the erythrocytic development [43,44]. This has been clearly confirmed for certain genes, such as *var *genes transcribed at the early ring stage and translated several hours later [45] or a series of sexual stage proteins the corresponding genes of which are transcribed long before translation occurs [46]. Genome mining revealed that relatively few genes (156, 2/3 less than expected) encode transcription associated proteins [17]. Six non-Zn finger transcription associated protein genes, as defined by Coulson [17], were over-expressed under artesunate, 3 were under-expressed (Table 8). This relatively low number suggests that transcriptional regulation may not play a major role in gene-expression modulation under drug-induced stress. Altered decay rates may modulate gene expression in drug-exposed parasites and contribute to the dynamic changes of mRNA levels observed here. In this regard, it is worth noting the recently outlined decreasing RNA decay rate as the parasite matures. A list of genes encoding putative decay components and possibly contributing to such a mechanism of post-transcriptional regulation in *P.falciparum *has been proposed [47]. However, there was no observed effect of artesunate on expression of these genes.

**Table 8:** Differentially expressed genes encoding transcription-associated proteins in artesunate-treated cells

**Gene ID**	**Description**	**Pfam**	**annotation**	**ART 90 min**	**ART 3 h**	**HS**	**CQ**
PF11_0053	PfSNF2L	TAP clustering	Nucleosome remodeling::SNF2L		0,82		
PF11_0264	DNA-dependent RNA polymerase	TAP clustering	MITOCHONDRIAL RNA POLYMERASE		1,02		
PFL0560c	minichromosome maintenance protein, putative	TAP clustering	Minichromosome maintenance protein		0,85		
PFE0090w	hypothetical protein, conserved	TAP clustering	Histone trancription regulator		0,82		
PF11_0297	hypothetical protein	TAP clustering	CCR4-NOT complex::NOT2		0,86	+	
PFE1245w	hypothetical protein, conserved	PF00642	Zinc finger C-x8-C-x5-C-x3-H type (and similar)(PF00642)	1,28	0,91	+	+
PF14_0236	hypothetical protein	PF00642	Zinc finger C-x8-C-x5-C-x3-H type (and similar)(PF00642)		0,89	+	
PFC0680w	hypothetical protein, conserved	PF00642	Zinc finger C-x8-C-x5-C-x3-H type (and similar)(PF00642)		1,55	+	
PF10_0186	hypothetical protein	PF00642	Zinc finger C-x8-C-x5-C-x3-H type (and similar)(PF00642)		0,98		
MAL13P1.122	hypothetical protein, conserved	PF00856	SET domain (PF00856)		0,81		
PFI0470w	FHA domain protein, putative	PF00097	C3HC4 type (RING finger) (PF00097)	0,89	1,05		
PF10_0046	hypothetical protein	PF00097	C3HC4 type (RING finger) (PF00097)		1,17		
PFF0165c	hypothetical protein, conserved	PF00097	C3HC4 type (RING finger) (PF00097)		0,94		
PFL0440c	hypothetical protein, conserved	PF00097	C3HC4 type (RING finger) (PF00097)		0,95		
PF11_0315	hypothetical protein	TAP clustering	APICOPLAST RNA beta, beta, & beta subunits		1,64		
PFL0145c	high mobility group protein	PF00505	HMG (high mobility group) box (PF00505)		-0,97		
MAL13P1.76	TFIIH basal transcription factor subunit	TAP clustering	TFIIH::p44		-0,83		
PF14_0718	hypothetical protein, conserved	TAP clustering	RNA polymerase II-associated factor SOH1		-0,86		

Gene ID: identifier as found in PlasmoDB.

Art columns: log ratios of gene expression under artesunate exposure at 90 minutes and 3 hours.

HS and CQ columns: over (+) expression.

Results were compared with transcriptome modifications induced by heat-shock (HS) [12] and chloroquine (CQ) [8].

Genes encoding transcription-associated proteins (TAPs) were selected from the TAP clustering of Coulson et al. [17] and completed with an update of genes fitting the following HMM profiles defined by Coulson and colleagues [16] : PF00097, PF00642, PF00096, PF00856, PF00249, PF00439, PF00505, PF00628, PF00098, PF00808, PF01753, PF00313, PF00569, PF00643, PF08523, PF02146, PF03366.

The low number of TAP identified by Coulson [17] contrasted with an over-representation of proteins with Zn finger motifs [48] potentially involved in regulating RNA stability. In the present study, 12 genes identified as putative Zn finger bearing proteins were differentially expressed in the presence of artesunate; 10/12 were over-expressed, two of which (PFI0470w and PFE1245w) already at 90 minutes (Table 9).

**Table 9:** Differentially expressed genes encoding proteins with Zn-finger motifs in artesunate-treated cells

**Gene ID**	**Description**	**Zn-finger**	**ART 90 min**	**ART 3 h**	**HS**	**CQ**
PFE1245w	hypothetical protein, conserved	Zinc finger C-x8-C-x5-C-x3-H type (PF00642)	1,28	0,91	+	+
PF14_0236	hypothetical protein	Zinc finger C-x8-C-x5-C-x3-H type (PF00642)		0,89	+	
PFC0680w	hypothetical protein, conserved	Zinc finger C-x8-C-x5-C-x3-H type (PF00642)		1,55	+	
PF10_0186	hypothetical protein	Zinc finger C-x8-C-x5-C-x3-H type (PF00642)		0,98		
MAL13P1.122	hypothetical protein, conserved	PHD-finger (IPR001965)		0,81		
PF14_0197	hypothetical protein	DNL zinc finger (PF05180)		0,87		
PFI0470w	FHA domain protein, putative	C3HC4 type (RING finger) (PF00097)	0,89	1,05		
PF10_0046	hypothetical protein	C3HC4 type (RING finger) (PF00097)		1,17		
PFF0165c	hypothetical protein, conserved	C3HC4 type (RING finger) (PF00097)		0,94		
PFL0440c	hypothetical protein, conserved	C3HC4 type (RING finger) (PF00097)		0,95		
PFB0140w	hypothetical protein	DHHC zinc finger domain (PF01529)		-0,91		
MAL13P1.76	TFIIH basal transcription factor subunit	TFIIH C1-like domain (PF07975)		-0,83		

Gene ID: identifier as found in PlasmoDB.

Art columns: log ratios of gene expression under artesunate exposure at 90 minutes and 3 hours.

HS and CQ columns: over (+) expression.

Results were compared with transcriptome modifications induced by heat-shock (HS) [12] and chloroquine (CQ) [8].

Genes containing Zn-motifs were defined as genes fitting the following Zn-finger HMM profiles: B-box zinc finger (PF00643), C3HC zinc finger-like (PF07967), C3HC4 type (RING finger) (PF00097), CHY zinc finger (PF05495), CSL zinc finger (PF05207), CW-type Zinc Finger (PF07496), DHHC zinc finger domain (PF01529), DNL zinc finger (PF05180), FYVE zinc finger (PF01363), HIT zinc finger (PF04438), MIZ/SP-RING zinc finger (PF02891), MYND finger (PF01753), PHD-finger (PF00628), Putative zinc finger motif, C2HC5-type (PF06221), Sec23/Sec24 zinc finger (PF04810), SWIM zinc finger (PF04434), TFIIH C1-like domain (PF07975), Tim10/DDP family zinc finger (PF02953), U1 zinc finger (PF06220), Zinc finger C-x8-C-x5-C-x3-H type (and similar)(PF00642), Zinc finger found in FPG and IleRS (PF06827), Zinc finger, C2H2 type (PF00096), Zinc finger, ZZ type (PF00569), ZN-finger, Zn-finger in Ran binding protein and others (PF00641), Zn-finger in ubiquitin-hydrolases and other protein (PF02148), ZPR1 zinc-finger domain (PF03367), AN1-like Zinc finger (PF01428), C2H2 and C2HC zinc fingers (SSF57667), PHD-finger (IPR001965).

## Discussion

The methodology adopted here allowed us to find reproducible alterations of the transcriptome in *P. falciparum *parasites under artesunate pressure. To overcome the major hurdle of developmentally regulated genes, we combined the use of a fast acting drug at high dose with limited incubation time at 5 successive but synchronized developmental stages. Most observed alterations were of low amplitude, possibly reflecting particular mechanisms of gene regulation in malaria parasites. This resulted in a relatively broad pertubation, that nevertheless was highly significant and consistent across experiments. We did not detect altered expression of a single specific metabolic pathway or of the putative drug target molecules but several functionally important groups of genes exhibited interesting dynamic changes. Within the intrinsic limitations of all transcriptome studies (in which steady-state RNA levels are studied and not protein quantities, post-translational modifications or biological activities), the overall picture that emerges from our current analysis of the transcriptome changes suggests i) pertubation of the intracellular trafficking and organisation, including changes in chaperones, transporters, remodeling the erythrocyte space beyond the parasitophorous vacuolar membrane up to the membrane of the infected erythrocyte; ii) probable down-phasing of metabolism as deduced from down-regulation of key enzymes/transporters implicated in purine, pyrimidine and isoprenoid synthesis; iii) an altered mitochondrion; iv) an altered redox homeostasis and possibly altered protein turnover. In addition, the transcriptome analysis highlighted numerous genes with unknown function, annotated as coding for hypothetical proteins [see Additional file 2]. Some display consistent high and early up-regulation and clearly deserve further investigation.

The physiological significance of the observed perturbations need to be assessed in future studies. Transcriptome analysis is the first of a multi-step process, to be followed by scrutiny of the role of the individual genes identified here. In particular, it will be essential to establish whether or not the genes in question are implicated in a rate-limiting process and to analyse the dynamics of the corresponding protein products, including their turnover, post-translational modifications and cellular localization. An important question is to clarifiy which transcriptome alterations are specific to the response to artemisinins and which are related to a parasite lethally injured, as this has numerous implications for drug development.

We will discuss the transcriptome data in the frame of existing knowledge on mechanisms of action of artemisinin derivatives and mechanisms resulting in reduced susceptibility/resistance. We will next discuss possible common changes in steady state RNA levels after different stresses inflicted to the parasite such as exposure to 41°C or treatment with chloroquine, that can cause parasite death.

The mechanism of action of artemisinins is debated. Artemisinin are endoperoxide-containing sesquiterpene lactones. Fe^++^-dependent activation of the endoperoxide bridge is required for the drug to be active [49]. Cleavage of the endoperoxide moiety forms highly reactive oxyl radicals that rearrange to more stable carbon-centered radical intermediates. These in turn form covalent adducts with parasite products thought to be responsible for a pleiotropic effect of the drug. Such pleiotropicity may explain the variety of genes we show here to be affected in their expression. Recent evidence suggest more specific mechanisms, such as inhibition of specific targets, with specific inhibition of the *P. falciparum *SERCA-type Ca++ pump (PfATPase6) [50]. Importantly, field isolates with markedly reduced *in vitro *susceptibility to artemether presented a mutant SERCA-type PfATPase6 [1,51]. Artemisinin has been shown to inhibit the endoplasmic reticulum – located SERCA-type in *Toxoplasma gondii*, a related Apicomplexan parasite as well [52]. We did not observe altered steady state levels of *PfATPase6 *mRNA at any time point investigated, indicating that over-expression of this gene is not part of the parasite response to artesunate.

Ultrastructural alterations of the morphology and their kinetics are debated as well. Early-stage alterations of the mitochondrion, the endothelial reticulum and the digestive vacuole were detected in some studies [53] but not others [54]. Some of the transcriptome alterations may account for a disorganisation of the digestive vacuole and the mitochondrion. We observed increased levels of *Pfmdr1 *expression concomitant with decreased mRNA levels of four of the subunits of the H^+ ^vacuolar ATPase, which is implicated in regulating calcium intracellular stores of acidic compartments. Downproduction of the H^+ ^vacuolar ATPase and/or alteration of its subunit ratio is predicted to negatively impact on intracellular calcium homeostasis. This is most probably on the critical path, since disruption of calcium homeostasis is central to cellular death, necrosis endophagy and apoptosis. Our transcriptome analysis also shows evidence that artesunate interferes with expression of genes related to the mitochondrion, in most cases by down-regulating their steady state RNA levels. This calls for additional studies on the mitochondrial activity after artesunate exposure.

Additional factors are to be considered in light of the transcriptome data, in particular the intracellular partitioning of the drug. Several studies have demonstrated the selective uptake of artemisinin derivatives. It has been shown that artemisinin and dihydroartemisinin, two sesquiterpene endoperoxide drugs closely related to artesunate, are transported by the tubulovesicular network (TVN) formed within the erythrocyte cytoplasm for nutrient import to the parasite, and that these drugs disrupted the protein organization of the TVN [55]. Numerous genes implicated in intracellular space modelling, trafficking, chaperones and transport display altered transcription profiles in artesunate-treated parasites. The markedly bias for such genes was indeed the most salient observation of our transcriptome study. It is tempting to speculate that this results in remodelling the intracellular space, including remodelling of the infected red blood cell membrane and TVN. This could either be a response aimed at correcting drug-inflicted damage, preventing further intake of drug or reflect the dysfunction resulting from such damage. Protein trafficking is all the more important for parasite growth and survival that the parasite develops inside a parasitophorous vacuole within the erythrocyte cytoplasm. Interactions between the parasite and the erythrocyte are multiple, with an increasing number of parasite proteins recognized as being involved (reviewed in [56]). Parasite-induced modifications of the red blood cell through interactions of parasite proteins exported to the host cell membrane or cytoskeleton play a major role in parasite survival and virulence, through induction of infected red cell cytoadhesive properties or decreased deformability. Altered transcript levels of genes involved in host cell remodelling and intracellular trafficking have been reported in parasites lethally damaged by exposure to febrile temperatures [12]. This suggests that perturbing intracellular trafficking/remodeling is either on the path to parasite death or an attempt to overcome a lethal injury. This also suggests that alterations of intracellular trafficking by artesunate may be one of the mechanisms through which the drug can be active on a wide range of parasite developmental stages.

Analysis of the proteome of artemether-treated *P. falciparum *parasites showed that 30 of the 101 proteins identified using a MALDI-TOF-MS-based analysis [57], displayed a greater than three-fold expression level compared to untreated control cultures. Two of the over-expressed proteins, namely hsp90 (PF07_0029), PF14_0425 (fructose-biphosphate aldolase) also presented an increased transcript level in our study. Direct comparison of both studies is precluded due to limitations such as different sensitivities of the transcriptome and proteome approaches, more comprehensive coverage of the expressed products in the transcriptome approach, different drug exposure protocols and/or transcription/translation uncoupling, which has been described for approx 50% of the proteins [43,44].

**Figure 3:**
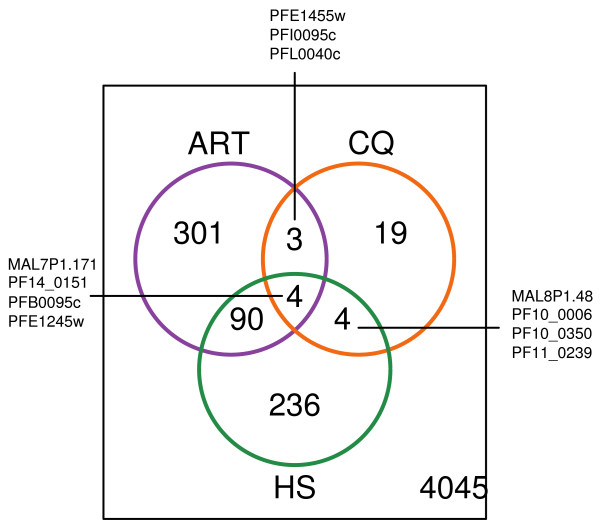
**Venn diagram of genes differentially expressed under artesunate treatment chloroquine treatment, and heat-shock**. Purple circle: 398 genes differentially expressed under artesunate (ART). Orange circle: 30 genes differentially expressed under chloroquine (CQ) [8]. Green circle: 334 genes differentially expressed under heat-shock (HS) [12]. ID of genes differentially expressed in ART and CQ, HS and CQ, ART, CQ and HS are shown.

To put the alterations observed under artesunate in perspective with changes induced by unrelated forms of stress, we analyzed the data in the light of the transcriptome alterations reported after chloroquine [8] treatment or incubation at 41°C [12], both treatments reported as having led to parasite death. The transcriptome studies are not directly comparable, as they were performed under different experimental conditions (synchronous/asynchronous parasites, glass slide microarrays/Affymetrix chips) and corresponded to different natures of stress (rapid acting vs.slow acting). However, identification of a few genes with altered expression under such different experimental conditions provides interesting insight. Such joint analysis is illustrated in the Venn diagram (Figure 3 see also tables 1, 2, 3, 4, 5, 6, 7, 8, 9, 10 for different functional groups). There were only 4 genes identified as differentially expressed under all 3 types of stress: PFB0095c encoding PfEMP3, PFE1245w, encoding a putative zinc finger protein, and PF14_0151 encoding a putative RNA-binding protein and MAL7P1.171 encoding a hypothetical protein. PfEMP3 and MAL7P1.171 have an export motif. PfEMP3 is exported to the cytoplasmic face of the erythrocyte membrane in the mature stages. It associates with the erythrocyte cytoskeleton, which it destabilizes [58]. Both PfEMP3 and MAL7P1.171 have been shown to contribute to trafficking of PfEMP1 [59,60]. Their role in the response to lethal stresses deserves to be investigated. Characterization of the products encoded by the remaining 2 common genes certainly warrants particular attention, as these genes may play a crucial role in the general response to stress.

The transcriptome of chloroquine-treated and artesunate-treated parasites shared three additional altered gene profiles, namely PFE1455w encoding a putative sugar transporter, PFI0095c and PFL0040c both encoding putative kinases belonging to the R45-FIKK family. Interestingly again, the three predicted proteins have an export motif. However, since there were large inter-experiment variations in the transcriptome analysis of chloroquine-treated parasites, commonalities are probably largely underestimated.

Ninety four genes were differentially regulated both under heat shock and artesunate, 63 over-expressed and 31 under-expressed under both conditions, amongst which members of the R45-FIKK kinase family, chaperones, molecules involved in lipid metabolism and number of hypothetical proteins -including as discussed above numerous genes coding for proteins with an export motif. Two of the 5 R45-FIKK kinase genes over-expressed under artesunate were also over-expressed under chloroquine pressure [8]. Of the 3 R45/FIKK kinase genes found over-expressed under heat shock, one was also differentially expressed under chloroquine. These data suggest that expression of genes belonging to the R45-FIKK kinase family reflects the response of the parasite to different types of environmental modifications and that the parasite response to different natures of stress may include common pathways. The same could be said about genes belonging to 2 other families. Eight of the 13 chaperone/chaperone-related encoding genes over-expressed under artesunate were also over-expressed under heat-shock. Of the 30 Hsp40 type *P. falciparum *chaperones, 6 were over-expressed under artesunate, 4 in common with heat-shock.

## Conclusion

The rapid parasiticidal activity of artesunate at high doses allowed us to study the effects of the drug on the transcriptome of synchronized parasite cultures over a short 3 hour time window. A positive bias was observed in favor of subtelomeric localization, polymorphism and presence of potential export sequences for the subset of upregulated genes. The corresponding genes, related to protein trafficking, kinases or membrane remodeling, often belong to multi-gene families [61]. Low amplitude transcript level alterations were observed, but the large number of affected genes likely induces substantial perturbations of the interface between the parasite and its host cellular environment. Whether these constitute parasite survival mechanisms or in contrast metabolic cytotoxic alterations remains to be established. The transcriptome analysis identified what may represent pathways leading to parasite death, such as inhibition of purine/pyrimidine metabolism, interference with the mitochondrial electron transport chain, with protein turn-over or with the integrity of the food vacuole and calcium stores.

The high proportion of over-expressed genes encoding proteins exported from the parasite highlight the importance of extra-parasitic compartments as fields for exploration in drug research which, to date, has mostly focused on the parasite within its plasma membrane rather than within its intra- and extra-erythrocytic environment.

## Methods

### Parasite cultures

The FCR3 strain (FUP/CB line) [62] of *P. falciparum *was used throughout the experiments. Parasites were cultured using the method described by Trager and Jensen with the modifications described in Ralph et al. [63]. Cultures were tightly synchronized by lysing maturing forms through treatment with 0.3 M Alanine at two successive cycles, starting 3 hours after rings appeared in the culture [63]. Artesunate treatment was performed the following cycle (see below).

### Drug treatment

Artesunate powder (Arenco, Belgium) was dissolved in PBS, and the 1 μg/μL aliquoted stock solution was kept at -20°C. To detect possible loss of activity during storage of stock solution, the IC_50 _of the drug was tested periodically. The IC_50 _remained stable at around 3.4 nM (2.2–4.6 nM, SD = 1.2). This also confirmed the artesunate sensitivity of the FCR3 strain.

Determination of minimal drug concentration irreversibly damaging 100% of the parasites: The lethal artesunate dose to be used throughout this investigation was determined using synchronized cultures incubated with increasing concentrations of artesunate (30-100-300-500-3000 ng/mL) for 3 hours, washed twice with RPMI and reincubated at 37°C for 24–30 hours, until next reinvasion. Reinvasion was assessed by examining Giemsa stained blood smears. The lethal dose was the minimal artesunate concentration needed to prevent 100% of reinvasion.

Parasite treatment for studies of the transcriptome: artesunate was added at five different time points during the cycle following parasite synchronization, staggered between 20 hours and 30 hours after appearance of the first rings in the culture (at approximately 20, 22, 26, 28 and 30 hours), while untreated cultures were cultured in parallel in the absence of drug. Parasites were extracted in Trizol after 90 minutes and 3 hours of incubation.

### Total RNA preparation

Parasite cultures were harvested by centrifugation and lysed in 5 pellet volumes of Trizol (Gibco) before freezing at -80°C. Total RNA was prepared from thawed samples following the manufacturer's instructions. RNA Quality was assessed with an Agilent 2100 Bioanalyser.

### Microarrays and hybridizations

The microarrays used here have been described elsewhere [63]. Briefly, glass slides were spotted with 7392 70-mer oligonucleotides originating from the Malaria Oligo Set (Qiagen-Operon) and custom design oligonucleotides, covering most of all *P. falciparum *genes. Of the 5542 identified *P. falciparum *genes, the array covers 4741 genes (85%). RNA labelling and hybridization were performed as described [63]. For the comparison of drug versus no drug at each time point, dye swaps with two technical replicates were performed to compensate dye effect and to assess technical reproducibility, leading to 4 hybridized slides per experiment.

In order to determine the genes differentially expressed between time 0 and 3 hours due to parasite development, three series of hybridizations were performed with cDNAs prepared at times 20/23 hours, 23/26 hours and 25/28 hours from the control cultures left without artesunate. Microarray data were deposited in ArrayExpress [ArrayExpress:E-MEXP-1435].

### Statistical analysis

All the slides were analyzed pooled together using R [64] and Bioconductor [65] with the limma package [66,67]. After logarithm transformation of ratio of median of the intensities (without background substraction) in the two channels, data were weighted to penalize spots of poor quality, flagged, or spots for which intensities in the two channels were close to background (median of intensity less than median of background plus 2 standard deviations for both Cy3 and Cy5 signals). In a second step, an intensity-dependent normalization was applied to each slide (loess), followed by quantile normalization [68] applied across the arrays to ensure that the log ratios had the same empirical distribution across arrays and across channels. After fitting a linear model to the expression data for each probe using the least square method, comparisons of interest were extracted with a contrast matrix. A moderated t-statistic and a log-odds of differential expression were computed for each contrast for each gene. Finally, the raw p-values were adjusted using the Holm procedure and a type I error rate of 0.05 was applied. When for a given gene significant log ratios were obtained for several oligos, the retained log ratio was one with the maximum absolute value.

### Reverse trancription and qPCR

RNA samples were treated with DNAse I (Invitrogen) for 15 minutes at 37°C in presence of RNAseOUT™ inhibitor (Invitrogen). RNA was reverse transcribed with Superscript II RT (Invitrogen) using random hexamers with pre-extension step of 10 minutes at 25°C followed by extension for 50 minutes at 42°C. Then, cDNA was treated with RNAse H for 20 minutes at 37°C.

Quantitative real time PCR was performed on cDNA using an ABI Prism 7900 HT thermal cycler system (Applied Biosystems) for 40 cycles (95°C for 15 s, 55°C for 15 s and 60°C for 45 s). Reactions were done in 20 μL volumes using SYBR Green PCR master Mix (Applied Biosystems) and 0.5 μM primers in triplicate with 6 concentrations of cDNA (2500 pg/μL, 625 pg/μL, 156 pg/μL, 39 pg/μL and 10 pg/μL) to assess primer amplification efficiency. Absence of DNA contamination was check using RNA sample treated as cDNA without RT (2500 pg/μL).

Primers were designed using eprimer3 [69] [See Additional file 5 for the primer sequences]. Transcript abundance was compared using mean of ΔΔCt values calculated for all cDNA dilutions using PFI0425w (putative transporter) as endogenous normalizer and the control condition as reference. This gene was chosen as it remained non differentially expressed in all our experiments and showed very moderate levels of variation in whole-cycle transcriptome analysis [4].

## Abbreviations

GSH: reduced glutathione; ACS: Acyl CoA synthetase; TAP: transcription associated protein; CQ: chloroquine; HS: heat-shock; ART: artesunate; SNP: single nucleotide polymorphism; HMM: hidden Markov model

## Authors' contributions

ON establishment of experimental protocoles, parasite cultures, drug exposure, RNA extraction, q-PCR, genome mining, interpretation of results, manuscript preparation. EB establishment of experimental protocoles, microarray spotting, q-PCR, genome mining, statistical analysis, interpretation of results, manuscript preparation. GD parasite cultures, drug exposure, RNA extractions, manuscript preparation. CP microarray spotting, hybridizations. MAD quality control of arrays, statistical analysis. OS microarray spotting, quality control of arrays. GG quality control of arrays, statistical analysis. SB interpretation of results, genome mining. JP interpretation of results. OMP interpretation of results, manuscript preparation. JYC spotting of microarrays, statistical analysis, interpretation of results. PHD establishment of experimental protocoles, parasite cultures, RNA extraction, genome mining, interpretation of results, manuscript preparation. All authors have read and approved the final manuscript.

## Supplementary Material

Additional File 1 **Distribution of log ratios for statistically significant genes**. Distribution of log ratios for genes differentially expressed upon 3 hour incubation with artesunate. Grey bars: genes selected (log ratio cut-off: +/- 0.8). Click here for file

Additional File 2 **Mean log ratio of gene expression in presence/absence of artesunate at 90 minutes and 3 hours**. List is made-up of genes displaying significant changes at 3 hour incubation time, across 5 experiments performed at time points staggered between 20–30 h of parasite development. Values shown express the mean log ratios calculated from all experiments; when for a given gene significant log ratios were obtained for several oligos, the retained log ratio was one with the maximum absolute value. In the 3 hour column, only values significant at 3 hours as defined by ANOVA are shown, with a mean log-ratio cut-off of +/- 0.8. Values in bold: log ratios of controls (0 hours vs 3 hours in absence of artesunate) have been substracted from the log ratios obtained after 3 hours in artesunate. Red cell-background: over-expression. Green cell-background: under-expression. Gene ID and description: from PlasmoDB. Click here for file

Additional File 3 **Confirmation by real-time quantitative rt-PCR of differential gene expression induced by 3 hour incubation with artesunate**. Transcripts abundances were compared by ΔΔCt values calculated using PFI0425w (putative transporter) as endogenous control. Values for microarray and qPCR are expression fold changes between parasites in presence and in absence of artesunate (Pearson correlation coefficient between the two techniques: 0.81). Click here for file

Additional File 4 **Genes related to cell cycle regulation**. Cell cycle associated genes were defined according to the GO biological process G0:0007049 (cell cycle). Red cell-background: over-expression. Green cell-background: under-expression. Click here for file

Additional File 5 Sequences of primers used for q RT-PCR validation. Click here for file
