# Supplementary figures and images for: Dynamic RNA profiling in Plasmodium falciparum synchronized blood stages exposed to lethal doses of artesunate
Source: BMC Genomics. 2008 Aug 18;9:388. doi: 10.1186/1471-2164-9-388 (PMC2536677; doi:10.1186/1471-2164-9-388)

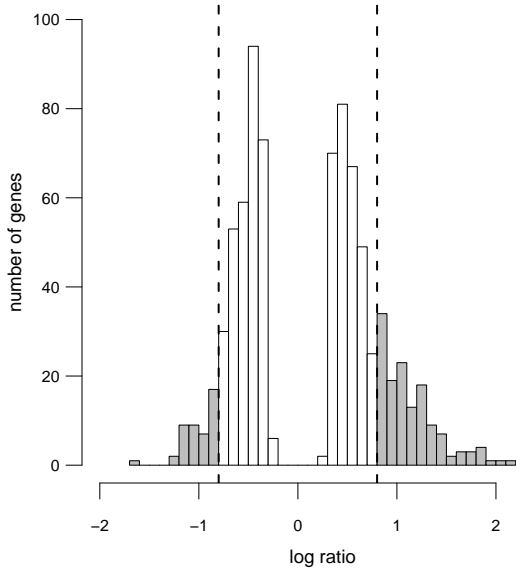

Supplement: Additional File 1 — Distribution of log ratios for statistically significant genes. Distribution of log ratios for genes differentially expressed upon 3 hour incubation with artesunate. Grey bars: genes selected (log ratio cut-off: +/- 0.8). [file 1471-2164-9-388-S1.pdf]
